# Patterns in blood pressure medication use in US incident dialysis patients over the first 6 months

**DOI:** 10.1186/1471-2369-14-249

**Published:** 2013-11-12

**Authors:** Wendy L St Peter, Stephen M Sozio, Tariq Shafi, Patti L Ephraim, Jason Luly, Aidan McDermott, Karen Bandeen-Roche, Klemens B Meyer, Deidra C Crews, Julia J Scialla, Dana C Miskulin, Navdeep Tangri, Bernard G Jaar, Wieneke M Michels, Albert W Wu, L Ebony Boulware

**Affiliations:** 1 University of Minnesota College of Pharmacy, Minneapolis, MN, USA; 2 Chronic Disease Research Group, Minneapolis Medical Research Foundation, 914 South 8th Street, Suite S4.100, Minneapolis, MN 55404, USA; 3 Division of Nephrology, Johns Hopkins University School of Medicine, Baltimore, MD, USA; 4 Welch Center for Prevention, Epidemiology, and Clinical Research, Johns Hopkins Medical Institutions, Baltimore, MD, USA; 5 Department of Epidemiology, Johns Hopkins Bloomberg School of Public Health, Baltimore, MD, USA; 6 Division of General Internal Medicine, Johns University Hopkins School of Medicine, Baltimore, MD, USA; 7 Department of Biostatistics, Johns Hopkins Bloomberg School of Public Health, Baltimore, MD, USA; 8 Division of Nephrology, Tufts University School of Medicine, Boston, MA, USA; 9 Department of Medicine, University of Miami Miller School of Medicine, Miami, FL, USA; 10 Department of Medicine, Division of Nephrology, Seven Oaks General Hospital, University of Manitoba, Winnipeg, Manitoba, Canada; 11 Nephrology Center of Maryland, Baltimore, MD, USA; 12 Division of Nephrology, Department of Medicine, Academic Medical Center, Amsterdam, The Netherlands; 13 Department of Health Policy and Management, Johns Hopkins Bloomberg School of Public Health, Baltimore, MD, USA; 14 Department of International Health, Johns Hopkins Bloomberg School of Public Health, Baltimore, MD, USA; 15 Department of Surgery, Johns Hopkins University School of Medicine, Baltimore, MD, USA

**Keywords:** Blood pressure medication, Dialysis, Medication use patterns

## Abstract

**Background:**

Several observational studies have evaluated the effect of a single exposure window with blood pressure (BP) medications on outcomes in incident dialysis patients, but whether BP medication prescription patterns remain stable or a single exposure window design is adequate to evaluate effect on outcomes is unclear.

**Methods:**

We described patterns of BP medication prescription over 6 months after dialysis initiation in hemodialysis and peritoneal dialysis patients, stratified by cardiovascular comorbidity, diabetes, and other patient characteristics. The cohort included 13,072 adult patients (12,159 hemodialysis, 913 peritoneal dialysis) who initiated dialysis in Dialysis Clinic, Inc., facilities January 1, 2003-June 30, 2008, and remained on the original modality for at least 6 months. We evaluated monthly patterns in BP medication prescription over 6 months and at 12 and 24 months after initiation.

**Results:**

Prescription patterns varied by dialysis modality over the first 6 months; substantial proportions of patients with prescriptions for beta-blockers, renin angiotensin system agents, and dihydropyridine calcium channel blockers in month 6 no longer had prescriptions for these medications by month 24. Prescription of specific medication classes varied by comorbidity, race/ethnicity, and age, but little by sex. The mean number of medications was 2.5 at month 6 in hemodialysis and peritoneal dialysis cohorts.

**Conclusions:**

This study evaluates BP medication patterns in both hemodialysis and peritoneal dialysis patients over the first 6 months of dialysis. Our findings highlight the challenges of assessing comparative effectiveness of a single BP medication class in dialysis patients. Longitudinal designs should be used to account for changes in BP medication management over time, and designs that incorporate common combinations should be considered.

## Background

The mortality rate for incident dialysis patients is high in the first few months. A large proportion of these deaths are due to cardiovascular causes, which remain the leading causes of death after the first 6 months [1]. Hypertension, congestive heart failure (CHF), and atherosclerotic heart disease occur in 85%, 32%, and 21%, respectively, of incident dialysis patients [1]. Two meta-analyses of small randomized clinical trials in dialysis patients receiving blood pressure (BP) medications suggest that BP treatment decreased cardiovascular events compared with control or placebo groups [2,3], particularly among patients with hypertension [2]. However, heterogeneity among the trials was substantial, and these analyses could not determine differential effects among medications. Data from observational studies suggest that exposure to specific medications (calcium channel blockers, renin-angiotensin system [RAS] agents, beta-blockers) is associated with reduced all-cause or cardiovascular mortality compared with no BP medication use [4-11]. However, clinical trials have been limited in their ability to conclusively support use of specific agents because of small sample sizes and heterogeneity of study designs. Observational studies to date have also been limited; some were conducted with prevalent patients with varying dialysis duration and comorbid conditions, possibly leading to problems with selection bias and confounding by indication. These concerns can be mitigated somewhat using incident populations. A more concerning issue relates to evaluation of associations based on exposure at one point in time. Understanding utilization patterns of BP medications in the months after dialysis initiation is imperative before appropriate designs for future studies can be determined.

However, there is a paucity of data regarding patterns of BP medication use in incident hemodialysis and peritoneal dialysis patients. Despite extensive use, no data examine longitudinal prescription patterns in the first few months of dialysis. To date, data are limited to cross-sectional analyses of prevalent [1,7,11-15] or incident dialysis patients [8,9,16]. In addition, these cross-sectional analyses have either focused on hemodialysis patients or have grouped all dialysis patients together, so very little information is available specifically regarding peritoneal dialysis patients. Of the studies in incident patients, two use data from 1996–1997 and do not reflect current practice patterns [8,9], and one was a single small hospital study and is not generalizable [16]. Changes in BP management over the first few months of dialysis, when residual kidney function may be present and volume status is changing, is also a key consideration.

Thus, a thorough understanding of BP medication prescription patterns among incident hemodialysis and peritoneal dialysis patients, accounting for common cardiovascular conditions that could influence prescription patterns after dialysis initiation, is critical to inform future comparative-effectiveness studies. We studied prescription patterns of single and combination BP medications over the first 6 months of dialysis in hemodialysis and peritoneal dialysis patients. We hypothesized that the average number of BP medications per patient would decrease, prescriptions by medication class would change over the first 6 months, and prescription patterns would vary by dialysis modality, sex, age, race, and cardiovascular disease (CVD) or risk factors.

## Methods

### Overview

Our study was part of the broader Developing Evidence to Inform Decisions about Effectiveness (DEcIDE) Network Patient Outcomes in End-Stage Renal Disease (DEcIDE-ESRD) Study funded by the Agency for Healthcare Research and Quality to examine the comparative effectiveness of common treatment strategies in ESRD [17]. Cohorts were selected from patients treated in Dialysis Clinic, Inc., (DCI) facilities 2003–2008. DCI is a not-for-profit medium-sized dialysis provider in the US with over 210 clinics in 27 states. DCI patient characteristics are similar to those of US dialysis patients in general, with an overrepresentation of black patients [1]. We linked DCI data to United States Renal Data System (USRDS) registry data to obtain additional information.

We constructed hemodialysis and peritoneal dialysis cohorts from all incident patients aged ≥ 18 years who initiated treatment January 1, 2003-June 30, 2008, in a DCI facility and were alive at 6 months, to examine BP medication patterns in the 6 months after dialysis initiation. We excluded patients whose DCI start date occurred more than 30 days after their USRDS start date (obtained from the Medical Evidence Report, Centers for Medicare & Medicaid [CMS] form CMS-2728), switched modalities (hemodialysis to peritoneal dialysis or vice versa, home hemodialysis, kidney transplant), transferred to a non-DCI facility, withdrew from dialysis, or were lost to follow-up during the first 6 months.

We determined presence of CVD, CHF, and diabetes at dialysis initiation from information on form CMS-2728 and over the first 6 months from Medicare claims in USRDS data using International Classification of Diseases Tenth Revision codes [17]. We determined oral BP medication prescriptions from medication lists in the DCI electronic medical record (EMR). This informational system maintains a history of all medications prescribed to each DCI patient, allows physicians to write prescriptions, and generates complete patient medication lists on care plans, physician encounter forms, and transfer summaries when patients are hospitalized or receive other outpatient care. The DCI EMR does not interface with inpatient or other (non-dialysis) outpatient clinic EMRs. Each month, patients bring in their medication bottles and nurses verify, reconcile, and record the medications to update the DCI EMR. BP medications are prescribed based on individual physician discretion.

We assessed medication use in each month based on drug start and stop dates in the EMR. We used the generic product identifier code to identify and classify each medication into specific antihypertensive classes using the Wolter’s Kluwer Medi-span® drug products database. We quantified the percentage of patients surviving to 6 months who were prescribed beta-blockers, RAS agents or dihydropyridine calcium channel blockers (DHP-CCB), and further quantified the percentage of surviving patients who continued to receive these classes of medications at months 12 and 24. We also assessed BP medication prescriptions in the most recent timeframe available (2007–2008) to determine the most current trends. Intravenous BP medications administered during dialysis sessions were not included.

### Statistical analyses

We described the cohorts, numbers of BP medications, and percentages of patients with prescriptions. We used descriptive statistics (mean ± standard deviation [SD]) for continuous variables (age, laboratory values) and percentages for categorical variables. We stratified the hemodialysis and peritoneal dialysis cohorts by modality, age, race/ethnicity, sex, CHF, CVD (atherosclerotic heart disease, cerebrovascular accident/transient ischemic attack, or peripheral vascular disease), and diabetes, as these variables have been shown to affect BP medication use [14]. We performed analyses using SAS version 9.2. We estimated all percentages to a precision with 95% confidence intervals of width ± 1.0% or less for hemodialysis and ± 3.3% or less for peritoneal dialysis patients.

The Johns Hopkins Medicine Institutional Review Board (Baltimore, Maryland) reviewed and approved the study.

## Results

**Figure 1:**
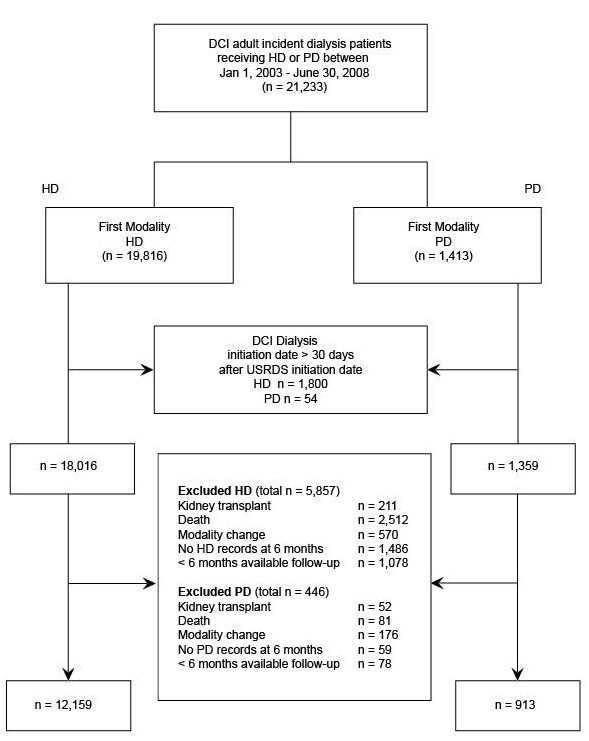
**Flow diagram for patients included and excluded from the study.** DCI, Dialysis Clinic, Inc; HD, hemodialysis; PD, peritoneal dialysis; USRDS, United States Renal Data System; patients were evaluated for exclusion criteria in the 6-month period after dialysis initiation.

After applying inclusion and exclusion criteria, our study cohorts consisted of 12,159 (93%) hemodialysis and 913 (7%) peritoneal dialysis patients, representing 62% of the initial cohort (Figure 1). Peritoneal dialysis patients were younger, less likely to be black, less likely to have ESRD caused by diabetes or hypertension, less likely to have cardiovascular conditions or diabetes, and more likely to have ESRD caused by glomerulonephritis; they had lower systolic BP and higher albumin and hemoglobin (Table 1).

**Table 1:** Patient characteristics at baseline

	**All**	**Hemodialysis**	**Peritoneal dialysis**
*n*	13,072	12,159	913
Age, years	61.6 ± 15	62.0 ± 14.9	56.0 ± 14.5
Sex			
Male	54.5	54.6	53.3
Female	45.5	45.5	46.7
Race			
White	58.8	58.2	66.5
Black	36.1	37.4	28.0
Other	4.5	4.4	5.5
Hispanic	5.4	5.6	3.6
ESRD cause			
Diabetes	48.1	48.5	43.4
Hypertension	27.3	27.7	21.7
Glomerulonephritis	9.5	9.0	16.9
Other	15.0	14.8	18.1
Cardiovascular risk factors or disease			
CHF	43.0	44.7	20.8
CVD	53.3	54.8	34.3
Diabetes	62.5	63.3	51.7
Other characteristics			
Systolic blood pressure	147.7 ± 20.2	147.9 ± 20.2	144.3 ± 19.5
eGFR	9.8 ± 4.4	9.8 ± 4.4	9.8 ± 4.1
Body mass index	28.7 ± 7.6	28.8 ± 7.7	28.4 ± 6.4
Albumin (g/dL)	3.1 ± 0.7	3.1 ± 0.7	3.6 ± 0.6
Hemoglobin (g/dL)	10 ± 1.7	10.0 ± 1.7	10.8 ± 1.5
Calcium x phosphorus	49.9 ± 13.0	50.1 ± 13.0	47.8 ± 13.3
KT/V	1.5 ± 0.3	1.5 ± 0.3	1.6 ± 0.4

*Note:* Values are mean ± standard deviation or percentages. Age, eGFR, albumin, body mass index, and hemoglobin were assessed at dialysis initiation; blood pressure was the average value for each patient over month 1; calcium x phosphorus and KT/V were the average value for each patient over months 4–6; cardiovascular risk factors or disease were assessed over the first 6 months.

CHF, congestive heart failure; CVD, cardiovascular disease; eGFR, estimated glomerular filtration rate; ESRD, end-stage renal disease.

### Mean number of blood pressure medications and prescription patterns, first 6 months

**Figure 2:**
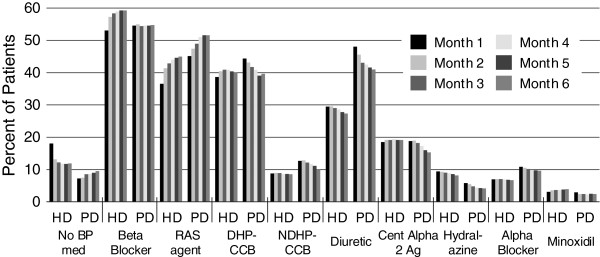
**Percentages of hemodialysis and peritoneal dialysis patients prescribed blood pressure medication classes or specific agents over the first 6 months after dialysis initiation.** BP, blood pressure; CCB, calcium channel blocker; Cent Alpha 2 Ag, central alpha 2 agonist; DHP, dihydropyridine; diuretic, any including thiazides, thiazide-like, loop, potassium-sparing; HD, hemodialysis; NDHP, non-dihydropyridine; PD, peritoneal dialysis; RAS, renin angiotensin system agent (angiotensin converting enzyme inhibitor or angiotensin receptor blocker).

Most patients with any recorded medications in the EMR were prescribed BP medications by 6 months after dialysis initiation (Table 2). The mean (± SD) number of prescribed medications was 2.8 ± 1.4 for peritoneal dialysis and 2.3 ± 1.4 for hemodialysis patients in month 1, but by month 6 the mean was 2.5 for both cohorts; thus the mean over the first 6 months of dialysis increased for hemodialysis and decreased for peritoneal dialysis patients. The percentage of peritoneal dialysis patients with no prescribed BP medications increased from month 1 to month 6, and the percentage of hemodialysis patients decreased slightly (Figure 2). Prescriptions for RAS agents increased in both groups. Prescriptions for DHP-CCBs remained stable for hemodialysis patients but declined for peritoneal dialysis patients. Diuretics were prescribed for 48% of peritoneal dialysis and 29% of hemodialysis patients at month 1. Diuretic prescription declined in both groups over the study timeframe. Central alpha 2 agonists (predominantly clonidine) were prescribed for 19% of hemodialysis and peritoneal dialysis patients in month 1; use of these agents declined for peritoneal dialysis patients by month 6. Minoxidil use was low in both groups**.** At month 6, the most highly prescribed classes were any beta-blocker (alpha-beta blockers such as carvedilol made up about one-third), any RAS agent (angiotensin-converting enzyme inhibitors [ACEIs] were twice as common as angiotensin receptor blockers [ARBs]), DHP-CCBs, diuretics, and central alpha 2 agonists.

**Table 2:** Percentages of incident hemodialysis and peritoneal dialysis patients with prescriptions for specific blood pressure medications at 6 months, by race/ethnicity, 2003–2008

	**Hemodialysis, *n *** **=12,159**					**Peritoneal dialysis, *n*** **= 913**				
**Medication**	**All**	**Black**	**White**	**Other**	**Hispanic**	**All**	**Black**	**White**	**Other**	**Hispanic**
*n*		4543	7075	541	679		256	607	50	33
None	12	10	13	13	11	10	8	10	xx	xx
Beta-blocker										
Any	59	60	59	58	55	55	54	55	56	42
Alpha-beta	19	20	18	16	14	13	14	13	xx	xx
Beta not alpha	41	40	41	42	41	42	40	42	44	30
RAS^*^										
Any	45	49	43	48	51	52	52	53	38	48
ACEI	32	36	29	33	39	33	32	34	20	xx
ARB	17	18	17	18	16	23	23	22	26	xx
CCB										
Any	49	57	43	49	55	50	55	37	50	45
DHP	40	48	34	43	48	40	48	35	48	39
Non-DHP	9	8	9	6	7	10	7	12	xx	xx
Diuretic^†^										
Any	28	23	31	29	33	41	32	45	42	45
Loop	26	21	29	26	32	38	31	42	36	42
Thiazide	5	4	5	5	5	9	6	10	xx	xx
Alpha blocker	7	7	7	4	5	10	10	10	xx	xx
Central alpha 2 agonist	19	27	15	12	19	15	21	14	xx	xx
Hydralazine	8	10	7	5	9	4	6	3	xx	xx
Minoxidil	4	6	3	3	2	2	5	2	xx	xx

*Note:* Values are percentages except for the *n* row, which represents numbers of patients.

ACEI, angiotensin converting enzyme inhibitor; ARB, angiotensin receptor blocker; CCB, calcium channel blocker; DHP, dihydropyridine; RAS, renin angiotensin system agent.

^*^Renin-inhibitors comprised < 1% of RAS agents from 2003–2008.

^†^Potassium-sparing diuretics comprised < 1% of diuretic agents from 2003–2008.

xx represents less than 10 persons per cell; these percentages have been suppressed.

### Patterns by demographic group

Black patients were prescribed a higher mean (± SD) number of BP medications than white or other-race patients (2.7 ± 1.4, 2.3 ± 1.3, 2.4 ± 1.3, respectively) at month 6. Prescriptions of any beta-blocker and of alpha-beta blockers were similar for black and white patients, but less for Hispanics. Prescription of RAS agents was similar for black and white peritoneal dialysis patients, but more frequent (particularly ACEIs) for black and Hispanic than for white hemodialysis patients. DHP-CCBs were less frequently prescribed for white hemodialysis and peritoneal dialysis patients than for other racial/ethnic groups, and loop diuretics for black patients. Alpha blocker prescriptions were similar for black and white patients, and central alpha 2 agonists, hydralazine, and minoxidil were more frequently prescribed for black than for white patients across modalities. Prescription information for other race and Hispanic ethnicity is provided for completeness, but comparisons between modalities may not be accurate for some medication classes due to few peritoneal dialysis patients in these groups (Table 2).

In general, older patients were prescribed fewer BP medications than younger patients; means at 6 months were 2.7 ± 1.4 for ages 18–44 years, 2.4 ± 1.3 for ages 65–74 years, and 2.2 ± 1.3 for ages ≥ 75 years. For hemodialysis patients, beta-blocker prescription was similar across age groups; RAS agent, DHP-CCB, central alpha 2 agonist, and minoxidil prescription declined monotonically as age increased, and alpha-blocker prescription increased monotonically. For peritoneal dialysis patients, beta-blocker prescription increased monotonically as age increased (46% for ages 18–44 years, 61% for ages ≥ 75 years), and RAS agent and DHP-CCB prescription varied across age groups. Prescription of central alpha 2 agonists and minoxidil decreased as age increased, but prescription of alpha blockers increased (data not shown).

Male and female hemodialysis patients were prescribed similar mean numbers of BP medications at month 6 (about 2.5), but female peritoneal dialysis patients received fewer prescriptions than male patients (2.4 vs. 2.7). Alpha blockers were prescribed more commonly for men (9% hemodialysis, 14% peritoneal dialysis) than for women (4% across modalities). Beta-blockers were prescribed more often for male than for female peritoneal dialysis patients (60% vs. 49; data not shown).

### Patterns by cardiovascular comorbidity and diabetes

BP medication prescription varied by cardiovascular comorbidity and cardiovascular risk factors. Any beta-blocker, alpha-beta blockers, and loop diuretics were more commonly prescribed for patients with CHF, CVD, and diabetes across modalities than for patients without these conditions. RAS agents were more commonly prescribed for patients with than without diabetes across modalities, but prescriptions were similar for patients with and without CHF or CVD. DHP-CCBs were less frequently prescribed for patients with CHF and CVD than for patients without these conditions. Hydralazine was more frequently prescribed for hemodialysis and peritoneal dialysis patients with than without CHF (Table 3).

**Table 3:** Percentages of incident hemodialysis and peritoneal dialysis patients with prescriptions for specific blood pressure medications at 6 months by cardiovascular risk factor or disease, 2003-2008

	**Hemodialysis, *n*** **= 12,159**							**Peritoneal dialysis, *n*** **= 913**						
		**CHF**		**CVD**		**Diabetes**			**CHF**		**CVD**		**Diabetes**	
**Medication**	**All**	**Yes**	**No**	**Yes**	**No**	**Yes**	**No**	**All**	**Yes**	**No**	**Yes**	**No**	**Yes**	**No**
None	12	10	13	11	13	10	15	10	7	10	7	11	5	14
**Beta-blocker**														
Any	59	65	55	64	53	62	54	55	69	51	67	48	61	48
Alpha-beta	19	26	13	22	15	20	16	13	25	10	17	11	16	10
Beta not alpha	41	39	42	43	38	42	38	42	44	41	51	37	45	38
RAS^*^														
Any	45	45	45	44	46	49	39	52	54	51	55	50	57	46
ACEI	32	32	31	31	32	34	27	33	36	32	34	32	35	30
ARB	17	16	17	16	18	19	14	23	22	23	25	22	26	19
**CCB**														
Any	49	44	52	45	52	49	47	50	43	51	48	51	54	44
DHP	40	36	43	37	43	41	39	40	35	41	38	41	42	37
Non-DHP	9	8	9	8	9	9	8	10	8	10	10	10	13	7
**Diuretic**^**†**^														
Any	28	31	24	28	26	31	21	41	55	37	52	35	50	32
Loop	26	30	22	26	24	29	19	38	53	34	50	33	47	29
Thiazide	5	5	4	4	5	5	3	9	13	8	13	6	12	5
Alpha blocker	7	6	7	7	6	7	7	10	8	10	12	9	11	7
Central alpha 2 agonist	19	18	20	17	21	20	18	15	14	16	14	16	16	14
Hydralazine	8	11	6	9	7	9	7	4	9	3	5	4	6	3
Minoxidil	4	3	4	3	5	4	5	2	2	3	1	3	2	2

*Note:* Values are percentages except for the *n* row, which represents number of individuals.

ACEI, angiotensin converting enzyme inhibitor; ARB, angiotensin receptor blocker; CCB, calcium channel blocker; DHP, dihydropyridine; RAS, renin angiotensin system agent.

^*^Renin-inhibitors comprised < 1% of RAS agents from 2003–2008.

^†^Potassium-sparing diuretics comprised < 1% of diuretic agents from 2003–2008.

### Patterns in combination blood pressure medications

**Figure 3:**
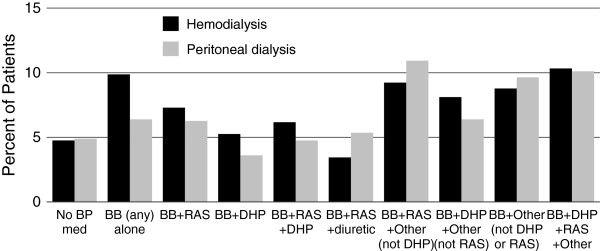
**Blood pressure medications (single or in combination) prescribed for ≥ 5% of hemodialysis or peritoneal dialysis patients with at least one blood pressure medication prescription.** BB, beta blocker; BP, blood pressure; DHP, dihydropyridine; diuretic, any including thiazides, thiazide-like, loop, potassium-sparing; RAS, renin angiotensin system agent (angiotensin converting enzyme inhibitor or angiotensin receptor blocker).

**Figure 4:**
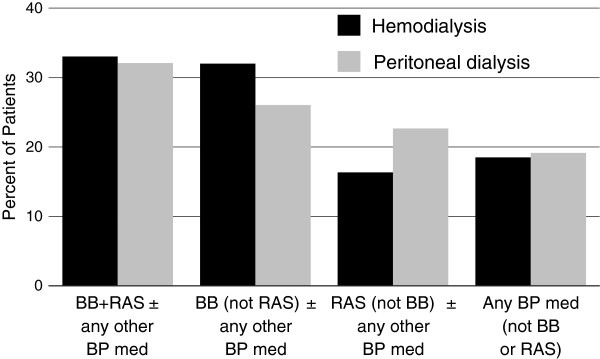
**Blood pressure medication combinations prescribed for ≥ 10% of hemodialysis or peritoneal dialysis patients with at least one blood pressure medication prescription.** BB, beta blocker; BP, blood pressure; RAS, renin angiotensin system agent (angiotensin converting enzyme inhibitor or angiotensin receptor blocker).

Combination BP medication prescriptions were common. Beta-blockers were the only single medication class prescribed in ≥ 5% of patients. In the overall cohort, 43 BP medication class combinations were detected with classes defined as any beta-blocker, any RAS agent, DHP-CCBs, diuretics, central alpha 2 agonists, and remaining categories “other”. Figure 3 shows the most common combinations prescribed for hemodialysis and peritoneal dialysis patients. As beta-blockers and RAS agents were overall the most frequently prescribed medications, when combinations were further collapsed to beta-blockers, RAS agents, and other, regimens including beta-blockers with and without RAS agents were the most common (Figure 4).

### Continuing blood pressure medications after 6 months

We evaluated the percentage of patients who survived and continued the same BP medication at 12 and 24 months as at 6 months. Of all patients prescribed a beta-blocker at 6 months, 89% and 76% of those who survived at 12 and 24 months, respectively, continued beta-blocker prescriptions. In comparison, 79% and 59% continued DHP-CCB prescriptions and 83% and 66% RAS agent prescriptions (data not shown).

### Patterns for 2007–2008

We evaluated the percentages of patients prescribed various BP medication classes and the most frequently prescribed agents in those classes at 6 months in the most current timeframe, 2007–2008, to evaluate trends over time. Compared with the overall timeframe of 2003–2008, prescription of beta-blockers across modalities and of ACEIs and loop diuretics in peritoneal dialysis patients were more frequent in 2007–2008. Percentages of patients prescribed ARBs, DHP-CCBs, alpha blockers, and central 2 alpha agonists remained stable (Table 4).

**Table 4:** Percentages of hemodialysis and peritoneal dialysis patients prescribed specific blood pressure medications within frequently prescribed classes,^* ^**2007–2008**

	**Hemodialysis patients**		**Peritoneal dialysis patients**	
**Class and medication**	**Class**	**Agent**	**Class**	**Agent**
Beta blockers	65.6		60.4	
Metoprolol succinate		25.5		32.8
Metoprolol tartrate		27.7		25.8
Carvedilol (alpha-beta blocker)		24.7		22.7
Atenolol		11.0		7.8
Labetalol (alpha-beta blocker)		9.1		7.0
% of class represented		98.0		96.1
ACEIs	31.4		39.6	
Lisinopril		66.6		60.7
Enalapril		9.7		1.2
Benazepril		9.5		16.7
Ramipril		4.6		7.1
Fosinopril		4.4		4.8
Quinapril		4.3		7.1
% of class represented		99.1		97.6
ARBs	17.0		23.1	
Valsartan		40.4		36.7
Losartan		31.4		38.7
Irbesartan		11.0		8.2
Olmesartan		9.2		4.1
Telmisartan		5.3		8.2
Candesartan		2.7		4.1
% of class represented		100		100
DHP-CCBs	43.4		39.2	
Amlodipine		66.2		61.4
Nifedipine		23.2		21.7
Felodipine		5.6		8.4
Nisoldipine		4.0		4.8
% of class represented		99.0		96.3
Loop diuretics	29.2		45.3	
Furosemide		84.7		89.6
Bumetanide		10.4		6.3
Torsemide		4.9		4.2
% of class represented		100		100
Central alpha 2 agonist	19.5		12.3	
Clonidine		98.9		96.2
Methyldopa		0.8		3.8
% of class represented		99.7		100
Alpha blockers	6.6		11.8	
Doxazosin		63.5		68.0
Terazosin		33.8		32.0
Prazosin		2.7		0.0
% of class represented		100		100
Hydralazine	11.1		4.7	
% of class represented		100		100

*Note:* Values are percentages.

ACEI, angiotensin converting enzyme inhibitor; ARB, angiotensin receptor blocker; DHP-CCB, dihydropyridine calcium channel blocker.

^*^Prescribed in at least 10% of hemodialysis or peritoneal dialysis patients at month 6 after dialysis initiation.

The most frequently prescribed beta-blockers in 2007–2008 were metoprolol succinate (sustained release), metoprolol tartrate (non-sustained release), and the alpha-beta blocker carvedilol. Lisinopril was the predominant ACEI prescribed, and losartan and valsartan the predominant ARBs. Amlodipine was the predominant DHP-CCB prescribed and doxazosin the predominant alpha blocker. Furosemide was virtually the only loop diuretic and clonidine the only central alpha 2 agonist prescribed.

## Discussion

Our study describes BP medication use in the first 6 months after dialysis initiation in a large population of hemodialysis and a smaller population of peritoneal dialysis patients. In hemodialysis patients, beta-blocker prescriptions increased over 6 months, DHP-CCB prescriptions remained stable, and diuretic and hydralazine prescriptions decreased slightly. In peritoneal dialysis patients, the percentage prescribed beta-blockers remained stable over 6 months and prescriptions of diuretics, DHP-CCBs, and central alpha 2 agonists steadily decreased. Prescription of RAS agents increased in both cohorts. The mean number of prescribed medications increased for hemodialysis but decreased for peritoneal dialysis patients over 6 months. Although prescription patterns appeared to stabilize in both cohorts by months 5 and 6, a substantial percentage of patients prescribed specific medications at month 6 were no longer prescribed those medications at month 24. Combination prescriptions were common in both cohorts. We found trends toward increased beta-blocker use in both cohorts and ACEI and loop diuretic use in peritoneal dialysis patients comparing 2003–2008 data with 2007–2008 data.

Our study has several strengths. First, data were obtained from the third-largest dialysis provider in the US, allowing us to evaluate differences in BP medication prescriptions between hemodialysis and peritoneal dialysis patients and to stratify our analyses by several characteristics, including baseline CHF, CVD, and diabetes, which influence prescription of cardioprotective medications [14]. Second, our analysis provides in-depth examination of contemporary prescribing patterns, which is important because BP medication prescription is influenced by available evidence on efficacy and by generic availability. Third, we evaluated use of combination prescriptions, which was not reported in previous studies.

Our results reflect a purely descriptive analysis; results have not been adjusted for important patient characteristics. In addition, our study did not identify reasons for apparent differences in prescription patterns by modality over the first 6 months. Some of the apparent differences may stem from differing baseline characteristics. For instance, beta-blockers were prescribed for more hemodialysis than peritoneal dialysis patients. Hemodialysis patients were older, on average, and beta-blockers were more likely to be prescribed for older patients. Hemodialysis patients had more CHF, CVD, and diabetes, and beta-blockers were more likely to be prescribed in patients with these conditions.

We hypothesized that the mean number of medications would decrease over time as volume control improved. However, the mean number of medications increased for hemodialysis patients but decreased for peritoneal dialysis patients over the first 6 months. Residual renal function declines more rapidly with hemodialysis than with peritoneal dialysis [18,19], possibly necessitating additional BP medications in hemodialysis patients over the first 6 months. For peritoneal dialysis patients, only prescription of RAS agents increased, and prescription of all other medications declined or remained stable (beta-blockers). Perhaps maintained residual renal function with RAS agents, along with better volume control, allowed for reduced medications in peritoneal dialysis patients.

Our findings regarding BP medication patterns among dialysis patients with CHF are consistent with Wetmore et al., who found that CHF was associated with a 9% increase in odds of beta-blocker use and no increase in RAS agent use in dually eligible (Medicare/Medicaid) prevalent hypertensive dialysis patients [14]. Our stratified analysis also showed much higher use of any beta-blocker in hemodialysis and peritoneal dialysis patients with than without CHF, driven mainly by prescription of beta-blockers with alpha activity. Similarly, we found little difference in RAS agent use among patients with or without CHF. Wetmore et al. also showed higher odds of RAS agent use in prevalent dialysis patients with than without diabetes [14], consistent with results from our stratified analysis.

In month 1, 48% of peritoneal dialysis and 29% of hemodialysis patients received diuretics, despite the same estimated glomerular filtration rate at dialysis initiation. However, diuretic use declined more rapidly over 6 months in peritoneal dialysis patients, which was unexpected since decline in renal function is slower in these patients [19]. Loop diuretics have not been shown to preserve residual renal function [20], and an interesting question relates to whether increased prescription of RAS agents in peritoneal dialysis patients after dialysis initiation led to decreased glomerular filtration rate or improved BP enough to obviate need for diuretics in some patients. One analysis of Dialysis Outcomes and Practice Patterns Study (DOPPS) data showed that diuretic use increased in incident US hemodialysis patients 1997–2001 (23% to 30%), and use was associated with lower interdialytic weight gain and lower odds of hyperkalemia [21]. Similarly, our 2007–2008 data showed diuretic prescriptions for 29% of DCI hemodialysis patients at month 6.

Comparing BP medication prescription data in our incident cohort of DCI dialysis patients in 2007–2008 with a large prevalent cohort of US dialysis patients enrolled in Medicare Part D in 2007 (*n* = 158,702), we found similar proportions prescribed beta-blockers (DCI: hemodialysis, 66%; peritoneal dialysis, 60% vs. USRDS: 64%), ARBs (DCI: hemodialysis, 17%; peritoneal dialysis, 23% vs. USRDS: 21%), and DHP-CCBs (DCI: hemodialysis, 43%; peritoneal dialysis, 39% vs. USRDS: 46%), but much higher proportions prescribed diuretics (DCI: hemodialysis, 29%; peritoneal dialysis, 45% loop diuretics vs. USRDS: 17% any diuretic), and lower proportions prescribed ACEIs (DCI: hemodialysis, 31%; peritoneal dialysis 40% vs. USRDS: 38%) and central alpha 2 agonists (DCI: hemodialysis, 20%; peritoneal dialysis, 12% vs. USRDS: 23%) [15]. The higher proportions prescribed diuretics in our cohorts presumably reflect higher residual renal function in the DCI incident than in the USRDS prevalent population.

Several observational studies using incident cohorts from Dialysis Morbidity and Mortality Wave 2 data have evaluated the influence of single BP or cardioprotective medications on long-term outcomes with exposure to a BP medication at a single point [6,8-10]; based on our findings, results may be confounded by substantial changes in BP medication prescription patterns over the first months of dialysis. We also found that a substantial percentage of patients prescribed specific medications at 6 months were no longer prescribed those medications at 24 months. This clearly requires further study. However, observational studies of BP medications in prevalent patients using a single exposure period and long follow-up periods may also be confounded [11].

Our findings regarding distributions of use for specific agents differed from those of a prior study of prevalent dialysis patients [14]. Regarding beta-blockers, we found higher use of metoprolol products and carvediol and less of atenolol and labetalol. As metoprolol succinate (sustained release) is now available in a generic version, one would expect prescription patterns to shift toward higher use if this study were repeated in a more contemporary timeframe. Carvedilol, a beta-blocker with alpha activity, reduces left ventricular volume in dialysis patients with dilated cardiomyopathy [22]. Generic carvediol became widely available in 2007, partially explaining its higher use in our cohort.

Prescription of multiple BP medications was common across modalities. Prescription patterns varied for each agent and by modality in the first 6 months after dialysis initiation. These patterns are important to consider in the design of comparative-effectiveness studies of BP medications in dialysis patients. As prescription patterns differ distinctly, hemodialysis and peritoneal dialysis patients should not be grouped together in studies evaluating the effect of medications on outcomes. On a population level, prescription patterns varied by medication class in the first 6 months, but appeared to stabilize by months 5 and 6. This suggests that a time-dependent approach should be used in assessing medication effect in incident patients if follow-up starts at dialysis initiation. Our evaluation of changes over time in individual patients prescribed beta-blockers, RAS agents, and/or DHP-CCBs at month 6 showed that time-dependent changes in prescriptions should be considered even after 6 months, particularly if follow-up time is longer than 6–12 months. Impact of and possible interactions with other concurrent medications should also be considered in designing future studies.

Our study has some limitations. Although characteristics of our cohort were similar to national characteristics [1], DCI medication prescribing patterns may not reflect patterns in other dialysis facilities. Since data were derived from EMRs, we know only that medications were prescribed, not whether patients consumed them. Further, accuracy of DCI outpatient medication lists may vary by dialysis unit and may not correctly reflect recent BP medication prescription changes made by nondialysis health care providers. In addition, we required patients to survive for 6 months in order to evaluate BP management in a stable cohort; in analyses of BP medications at 12 and 24 months, we required patients to survive to those points. Prescription patterns may differ for surviving patients and for patients who died. The number of included peritoneal dialysis patients was relatively small, which limited interpretation of results in some race and ethnicity groups. Finally, although we stratified our findings by important patient characteristics, our analyses are largely descriptive and do not adjust for multiple characteristics that could influence medication prescription. Thus, the data from hemodialysis and peritoneal dialysis patients are not directly comparable, because the characteristics of these populations differ.

## Conclusions

In conclusion, our study evaluates BP medication prescription patterns in hemodialysis and peritoneal dialysis patients in the first 6 months after dialysis initiation. Observational studies attempting to compare medication effectiveness should account for the time-varying nature of BP medication prescriptions, particularly in the first 6 months and if follow-up extends beyond 6–12 months. Given that most patients are prescribed multiple medications, investigators should consider study designs that incorporate medication combinations. Finally, prescription patterns after dialysis initiation differ by modality, and study designs should account for these differences. Further research is necessary to determine the effects of BP medication patterns on intermediate outcomes such as BP variability, and key clinical outcomes such as cardiovascular mortality and hospitalization, sudden cardiac death, and quality of life.

## Competing interests

The authors report no conflicts of interest with the study subject matter.

## Authors’ contributions

All authors participated in study conception or design, or analysis and interpretation of data, or both; Dr. St. Peter drafted the manuscript and the other authors provided intellectual content of critical importance to the work. All authors approved the version to be submitted.

## Pre-publication history

The pre-publication history for this paper can be accessed here:

http://www.biomedcentral.com/1471-2369/14/249/prepub
